# Supplementary material for: Pesticide Exposure of Residents Living in Wine Regions: Protocol and First Results of the Pestiprev Study
Source: Int J Environ Res Public Health. 2023 Feb 22;20(5):3882. doi: 10.3390/ijerph20053882 (PMC10001537; doi:10.3390/ijerph20053882)

Supplementary material

TABLE OF CONTENTS

1 Pesticides products 2
2 Pesticide analysis procedure 2
2.1 Chemicals and materials 2
2.2 Extraction procedure 3
2.2.1 Wipe Extraction 3
2.2.2 Hand washing water extraction 3
2.3 Analytical procedure 3
2.3.1 HPLC–MS/MS 3
2.4 QA and QC 4
2.4.1 Blanks 4
2.4.2 Matrix reference material and absolute recoveries 4
2.4.3 Limits of quantification 4
2.4.4 Validation of analysis 5
3 Pesticide quantification frequencies and median concentrations of quantified pesticides by type of sample 6

1 Pesticides products

Table S 1: Selected pesticides along with physico-chemical properties and ecotoxicological values.

Name	Pesticide type	Substance group	CAS	Approval status*	Banned date	DT50[a]	DT90[a]	Log Kow
**Benalaxyl**	Fungicide	Acylamino acid	71626-11-4	Not approved	2021	66.8	222	3,54
**Boscalid**	Fungicide	Carboxamide	188425-85-6	Approved		254	1000	2,96
**Cymoxanil**	Fungicide	Acetamid	57966-95-7	Approved		3.5	-	0.67
**Pyraclostrobin**	Fungicide	Strobilurin	175013-18-0	Approved		33.3	234	3,99
**Tebuconazole**	Fungicide, Plant growth regulator	Triazole	107534-96-3	Approved		47.1	177	3,7
**Trifloxystrobin**	Fungicide	Strobilurin	141517-21-7	Approved		1.69	20.9	4,5

[a] *field*

2 Pesticide analysis procedure

2.1 Chemicals and materials

Solvents were HPLC-grade quality. Acetonitrile (JT Baker), Methanol (JT Baker), and ammonium acetate (5 mM pH7 JT Baker) were supplied by Atlantic Labo (Eysines, France). The acetic acid was 99.7% purity (Sigma-Aldrich, St Quentin Fallavier, France). Milli-Q grade water was prepared from a Milli-Q system (Millipore SA, St Quentin les Yvelines, France) according to the following criteria: total organic carbon < 2 ppb, resistivity 18,2 MΩ at 20 °C. All the pesticides and internal standards were of a high purity grade (purity > 98%) from Cluzeau Info Labo (Ste Foy la Grande, France). Nitrogen gas was > 99.9995% purity, supplied by Messer (France SAS, Carbon Blanc, France).

The summary of the optimized parameters HPLC–MS/MS defined for each substance is presented in Table S2. For analyzed compounds, a high-performance liquid chromatography separation was realized with an HPLC Infinity 1290 using a C18 kinetex column (1.7µm C18 100A 100x2.1mm Phenomenex, Torrance CA, USA). The detection was performed using a triple quadrupole 6495B from Agilent Technologies (Santa Clara, CA, USA). After separation, the compounds were ionized with an electrospray-type source in positive mode. Nitrogen was the collision gas, allowing analysis in tandem mass spectrometry in dynamic MRM mode. Two transitions were used to identify each substance: a quantifier transition (QT1) from the fragmentation of the precursor ion into the product ion and a qualifier transition (QT2) from the product ion. The ratio of quantifier and qualifier transitions QT1/QT2 should remain the same and is a part of the validation method.

Table S 2: Optimized conditions for HPLC–MS/MS analysis with acetic phase including the quantifier transition (QT1) and qualifier transition (QT2), the Collision cell exit potential (CE), Retention time (RT), Ionization mode and ratios of QT1/QT2. Isotopically labeled standards are reported in purple.

ACETIC ACID PHASE							
**Substances**	**Quantifier transition (QT1)**	**CE QT**	**Qualifier transition (QT2)**	**CE CT**	**RT (min)**	**Ionization mode**	**QT1/QT2**
**Atrazine d5**	**221.1->179.0**	**16**	**-**	**-**	**8.43**	**+**	
Benalaxyl	326.1->148.1	20	326.1->91.1	54	11.09	+	1.1
Boscalid	343.0->306.9	16	343.0->139.9	16	9.79	+	4.3
Cymoxanil	221.1->175.9	4	221.1->149.9	4	5.56	+	1.9
Pyraclostrobin	388.0->194.0	10	388.0->163.1	25	11.32	+	1.4
Tebuconazole	308.2->70.1	25	308.2->125.0	45	10.94	+	13.5
**Tebuconazole d6**	**314.19->71.8**	**16**	**-**	**-**	**10.9**	**+**	
**Terbutylazine d5**	**235.0->179.0**	**12**	**-**	**-**	**9.63**	**+**	
Trifloxystrobin	409.1->186.0	12	409.1->145.0	52	11.78	+	1.4

2.2 Extraction procedure

Wipe and hand washing water samples were stored at -20°C as per the previous extraction procedure. Two different methods were developed depending on the matrix type (i.e., wipe and hand washing water).

Commented [WP1]: Please check meaning retained.

Commented [RT2R1]: I confirm

2.2.1 Wipe Extraction

The extraction was conducted on a whole wipe. First, a mixture of isotopically labe~~l~~led standards previously prepared in methanol was added to the sample. Two sequential extractions were performed by adding 2 x 50mL of acetonitrile into a 100mL glass bottle. The ultrasonic extraction (VWR) was conducted ~~during~~ over 15 min. An amount of 10mL of the final extract solution was evaporated under nitrogen flux until a 200µL final volume was reached.

2.2.2 Hand washing water extraction

For hand washing sample water, a mixture of isotopically labeled standards previously prepared in methanol was added to the 1mL sample. Then, 120µL was directly injected and analyzed by LC/MS/MS (Dynamic MRM mode).

2.3 Analytical procedure

2.3.1 HPLC–MS/MS

An HPLC Infinity 1290 from Agilent Technologies (Santa Clara, CA USA) was used coupled to a quadrupole mass spectrometer 6495B from Agilent Technologies. The column used to separate the substances after the 5 µL injection was a Kinetex column (100 x 2.1 mm ; 1,7 µm, Phenomenex, Torrance CA, USA) with a reverse phase C18 kept at 45°C and a mobile phase gradient (0.5 $mL.min^{-1}$) adapted to the analytical method (Table S 3).

Commented [WP3]: Please check meaning retained.

Commented [RT4R3]: I confirm

Table S 3: Mobile phases used in the column of HPLC–MS/MS

Mobile phase	A	B
Acetic acid phase	Milli-Q water + 0,1 % acetic acid + 5mM ammonium acetate	Methanol

During analysis, the gradient changes from a 100% aqueous phase to a 100% organic phase in 14 min. The gradient changes back to the 100% aqueous phase in 2 min to condition the system before the next injection. After separation, the substances were ionized by an electrospray source in positive mode. Nitrogen was used in the collision cell to conduct the mass spectrometry analysis with the dynamic mode MRM.

2.4 QA and QC

2.4.1 Blanks

A blank sample was conducted for every series of samples prepared to identify potential contamination during extraction steps. The blanks went through the entire analytical process. Four substances were affected by contamination issues: one of them was concerned by one-time blank contamination (benalaxyl), whereas recurring contaminations were noticed for the others. For those late substances, the HPLC instrument residual contaminations were identified as responsible for the blank contamination. To avoid any false quantification, the concentrations measured in the blank samples were deducted from the wipe and hand washing sample concentrations. Blanks were also used to calculate limits of quantification when necessary (see below).

2.4.2 Matrix reference material and absolute recoveries

An artificial spiked wipe or mineral water (Vittel, France) with the target compounds was used in each batch of sample extractions to control potential matrix effects during extraction and ensure correct recoveries. The wipe and hand washing water reference material consists of wipe samples or mineral water, with no pesticide contamination, spiked with a mixture of isotopically labeled standards and native substances at known concentrations. The spiked samples were then extracted following the protocol described above.
The quality of substance recovery after extraction was assessed by calculating recoveries as follows:

$$Recovery_{\%} = \frac{C_{measured} - Ci}{C_{spiked}} \quad \text{(a)}$$

where $C_{measured}$ is the final concentration of the spiked substances measured in the matrix reference material; Ci is the concentration measured in the unspiked matrix; and C_{spiked} is the spiked concentration.

The substances presented recoveries ranging from 80 to 110% with variabilities below 20% for the wipe matrix and from 92% to 105% with variabilities below 15% for hand washing water (Table S4).

2.4.3 Limits of quantification

Two types of limits of quantification were calculated and combined to determine a final LOQ for each substance. A limit of quantification was calculated from the matrix reference material spiked (wipe or mineral water) with known concentrations. For each substance, the ratio of the amount of injected substance reported to the initial extracted sample must exhibit a signal-to-noise ratio (S/N) of at least over 10. The noise zone used to quantify the

S/N value is usually selected just before the peak. Limits of quantification were validated using a batch of matrix reference samples spiked at the concentration identified as LOQ. Parameters of the analysis (quantifier and qualifier transitions, QT1/QT2, S/N ratio) were verified to validate the limit of quantification.

The final limits of quantification were then compared to blank concentrations multiplied by 10 (Blank-LOQ). The highest concentration between Blank-LOQ and the final LOQ was chosen as consolidated LOQ. The final consolidated limits of quantification are reported in Table S4.

Table S 4: Recoveries and limits of quantification for all the substances.

**Substance**	**Extraction recovery (%) wipe**	**Final limit of quantification (ng per wipe) wipe**	**Extraction recovery (%) Hand washing water**	**Final limit of quantification (ng L^{-1}) Hand washing water**
**benalaxyl**	90	**0.06**	**96**	**0.2**
**boscalid**	102	**0.06**	**99**	**8.0**
**cymoxanil**	83	**1.00**	**105**	**60.0**
**pyraclostrobin**	101	**1.50**	**93**	**0.4**
**tebuconazole**	106	**0.05**	**97**	**0.7**
**trifloxystrobin**	110	**0.02**	**100**	**0.5**

2.4.4 Validation of analysis

Instrumental precisions and performances were verified before every batch of matrix (wipe and hand washing water) analysis using a mixture of native substances and isotopically labeled standards at known concentrations. Those calibration curves obtained were used to calculate the concentrations of substances in the samples. Quantification recoveries were therefore verified by comparing calculated concentrations after analysis based on calibration curves and initial concentrations. As internal-labeled standards and native substances might have a slight difference in responses, a response coefficient (Ki) was calculated before every sequence of analysis to correct the variations between ILS and substance:

$$Ki = \frac{m_x \text{ x } A_{ILS}}{m_{ILS} \text{ x } A_x} \quad \text{(b)}$$

Ki: response coefficient of the substance compared to its associated internal-labeled standard

m_x: mass of the substance x in the mixture solution

A_{ILS}: internal-labeled standard area

m_{ILS}: mass of the internal-labeled standard in the mixture solution

A_x: area of the substance x

From this response coefficient, the mass of each substance can be calculated using the following equation:

$$m_x = \frac{Ki \text{ x } m_{ILS} \text{ x } A_x}{A_{ILS}} \quad \text{(c)}$$

mx: mass of the substance of interest in the sample

Ki: response coefficient of the substance compared to its associated internal-labeled standard

m_{EI}: mass of internal-labeled standard added to the sample before extraction

Ax: area of the substance of interest

$\mathcal{A}_{EI}$: area of internal-labeled standard

A long-term quality control of blanks and calibration has been conducted by the laboratory to assess the stability of analytical quality in order to identify potential anomalies and validate analytical series. In addition, the comparison of the areas of the internal-labeled standard obtained for matrix samples and spiked matrix reference material was automatically performed to identify potential additional matrix effects of specific samples (wipe and hand washing water).

3 Pesticide quantification frequencies and median concentrations of quantified pesticides by type of sample

Figure S1 – Pesticide quantification frequencies and median concentrations of quantified pesticides by type of sample for the three houses included in the PESTIPREV study.

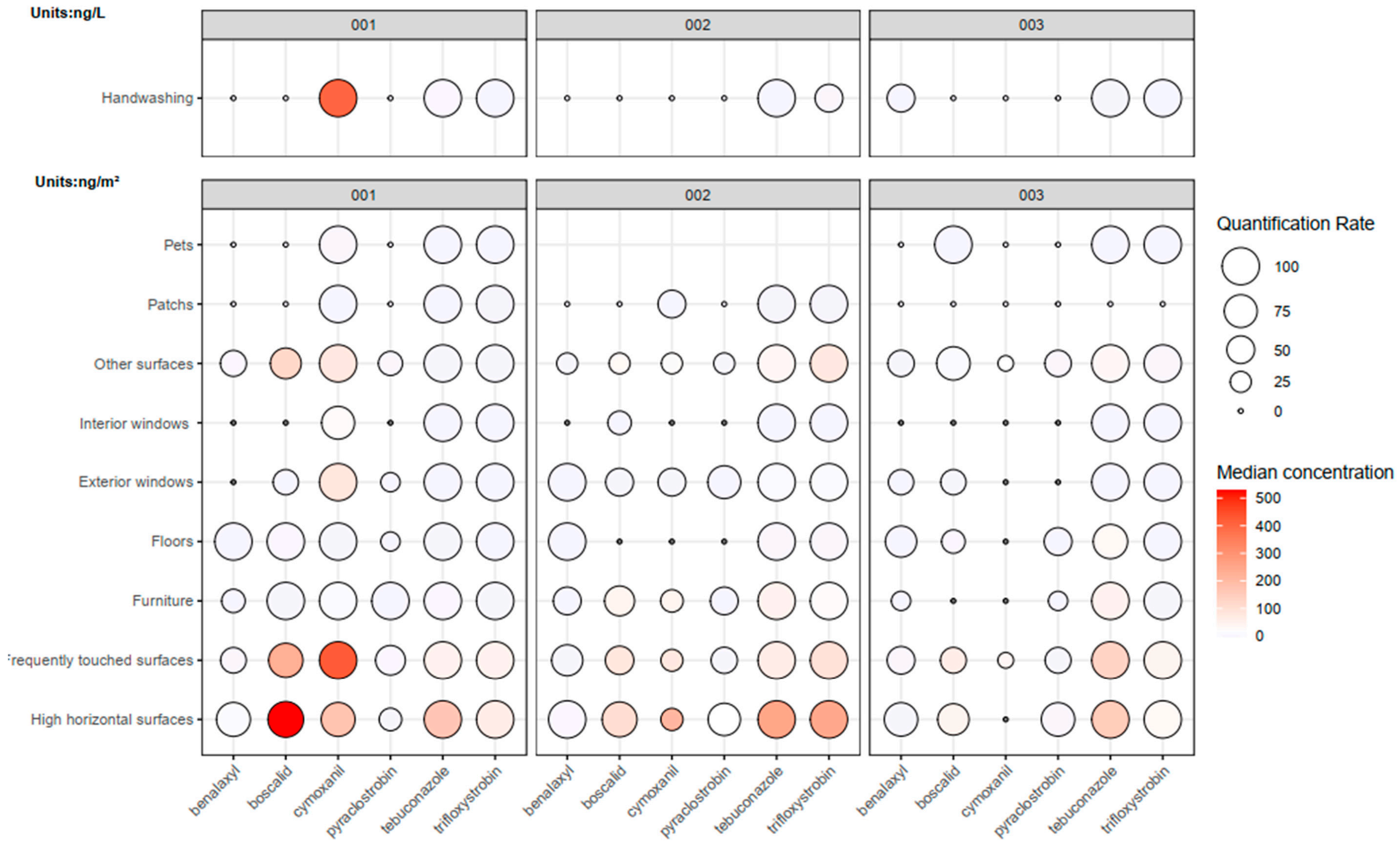

Supplement: Supplementary file 1 [file ijerph-20-03882-s001.zip › ijerph-2181329-supplementary.pdf]
